# Cesarean section rates in Brazil: Trend analysis using the Robson classification system

**DOI:** 10.1097/MD.0000000000019880

**Published:** 2020-04-24

**Authors:** Edson Luciano Rudey, Maria do Carmo Leal, Guilhermina Rego

**Affiliations:** a Faculty of Medicine of Porto University, Porto, Portugal; b Oswaldo Cruz Foundation (FIOCRUZ), Rio de Janeiro, Brazil.

**Keywords:** Brazil, cesarean section, Robson classification

## Abstract

Cesarean section (CS) is a surgical procedure used to deliver babies that is medically indicated to prevent maternal and neonatal mortality. However, it is associated with short- and long-term risks. CS rates have increased, and efforts are being made to ensure that CS is performed only when necessary. The Robson classification system is considered useful for studying, evaluating, monitoring, and comparing CS rates within and between healthcare facilities. In Brazil, there are few studies on this subject, and no large epidemiological studies on this topic utilizing the Robson classification have been reported. This study aimed to report and analyze CS rates in Brazil using the Robson classification system, and subsequently suggest possible measures to address it.

Data were collected from the Brazilian Live Birth Information System (Sistema de Informações sobre Nascidos Vivos) that contains data of the entire obstetric population, from 2014 to 2017. All births in the country during this period were analyzed according to the Robson classification.

A total of 11,774,665 live births were reported in Brazil during 2014 to 2017, most of which were mostly via CS (55.8%). Regions with high human development indexes had significantly higher CS rates than those with low human development indexes. The Robson group (RG) 1 to 4 accounts for 60.2% of live births and 47.1% of all CSs. RG5 was larger than all the other groups and contributed to the highest global rate of CS (31.3%), in addition to being the group who presented the largest growth.

Although RG 1 to 4 present favorable initial conditions for vaginal delivery, CS accounted for almost half of births in these groups. The size of RG1 and RG2 in Brazil was comparable to that in countries with low CS rates; however, CS rates in these groups were 3 times higher in Brazil. Nulliparous women in RG1 and RG2 who undergo CS are subsequently categorized into RG5, increasing the global CS rate by 1% annually.

We suggest the implementation of health policies to avoid the unnecessary performance of CS in RG1 and RG2 to decrease the CS rates in Brazil.

## 1 Introduction

Cesarean section (CS) is a surgical procedure medically indicated to prevent maternal and neonatal mortality during childbirth.^[1,2]^ However, similar to other surgeries, it is associated with short- and long-term risks that may persist for many years thereafter, affecting the health of both the mother and child. Short-term risks of CS include the increased probability of neonatal respiratory distress and increased rates of maternal blood transfusion, organ damage, thromboembolic diseases, anesthetic complications, and infections. The long-term risks involve increases in the risk of complications in subsequent pregnancies, such as placenta accreta and uterine rupture, as well as the risk of obesity and asthma in children.^[3–7]^

Despite these risks, CS rates have increased worldwide over the past few decades^[8–10]^ regardless of the diversity in the access to healthcare services.^[11]^ The mean global CS rate is 18.6%, with the lowest and highest rates found in Africa (7.3%) and South America (42.9%), respectively.^[11]^ In Brazil, the CS rate was 55.9% in 2018.^[12]^

Determining an appropriate CS rate and defining the situations in which CS is indicated are major challenges; nevertheless, these are required to avoid unnecessary surgery.^[13]^ Systematic reviews performed by the World Health Organization (WHO) concluded that CS rates in the population of up to 10% to 15% are associated with decreased neonatal and maternal mortality rates.^[14]^ However, when the CS rates exceed 10%, it was not associated with decreased mortality.^[13]^ Even in more developed countries, the increase in CS rate does not significantly decrease the maternal and neonatal mortality.^[15]^ Moreover, there is a greater risk associated with unnecessary CS in those of low socioeconomic status because poorer regions usually provide limited access to good quality obstetric care and safe facilities.^[1]^ Rates of maternal and perinatal deaths after CS are disproportionately high in low- and middle-income countries; for example, the rate of maternal deaths in the sub-Saharan region is 100 times higher than that of the United Kingdom.^[16]^ In Brazil, CS is considered an independent risk factor for postpartum maternal death.^[17]^

There are several causes for increased CS rates. Changes in population characteristics, including an increased number of older nulliparous pregnant women and obese pregnant women, are considered contributing factors to increased CS rates.^[1]^ The study “Nascer no Brasil” (Being Born in Brazil), which investigated the determinant factors and magnitude of obstetric interventions, including unnecessary CS, concluded that there is no clinical basis for such a high percentage of surgeries.^[18]^ Therefore, factors such as varying professional practices, economic, social, and cultural factors, and increased fear of medical litigation have increased the CS rates.^[19–21]^

The WHO states that efforts should be made to ensure that CS is performed only when necessary, rather than defining a specific rate,^[13]^ and proposes the use of the Robson classification system^[22]^ worldwide to evaluate, monitor, and compare CS rates both within and between healthcare facilities.^[13]^ By recommending the Robson classification, the WHO initiated a uniform data collection process that can be used in further studies on CS rates.^[23]^ In Brazil, there are few studies on the subject, and no large epidemiological studies that utilize the Robson classification have been reported to date.

The main objective of this study was to report and analyze CS rates in Brazil from 2014 to 2017 using the Robson classification system and suggest possible measures to address the issue of high CS rates. The secondary objective was to compare CS rates between regions with a high and low human development index (HDI) according to the Robson classification system.

## 2 Methods

This study was based on secondary data collected from the website of the Department of Epidemiological Information and Analysis of the Federal Health Surveillance Secretariat.^[12]^ The Live Birth Information System (Sistema de Informações sobre Nascidos Vivos [SINASC]) was officially implemented in 1990 to collect birth data throughout the country and provide information on birth rates at all levels of the healthcare system. This system collects information from the Certificate of Live Birth, a standard mandatory document in Brazil.^[12]^

The study included all live births (LBs) in Brazil registered in the system from 2014 to 2017. Two groups were formed; one for states and the other for cities, and these were subdivided based on the HDI level.

### 2.1 Robson groups (RGs) and covariates

The research variables included the total number of LBs, number of CS births, and Robson classification groups, as explained on the website of the Department of Epidemiological Information and Analysis of the Federal Health Surveillance Secretariat, which are as follows^[12]^: RG1 (nulliparous women with a single cephalic pregnancy at ≥37 weeks’ gestation in spontaneous laborRG2 (nulliparous women with a single cephalic pregnancy and ≥37 weeks’ gestation with induced labor or delivery through CS), RG3 (multiparous women [no history of CS] with a single cephalic pregnancy and ≥37 weeks’ gestation in spontaneous labor), RG4 (multiparous women [no history of CS] with a single cephalic pregnancy and ≥37 weeks’ gestation with previous induced labor or CS delivery), RG5 (previous CS with a single cephalic pregnancy and ≥37 weeks’ gestation), RG6 (all nulliparous women with pelvic delivery), RG7 (all multiparous women with pelvic delivery [including previous CS delivery]), RG8 (all women with multiple pregnancies [including previous CS delivery]), RG9 (all women with abnormal lie presentation during delivery [including previous CS delivery]), and RG10 (all women with a single cephalic pregnancy and <37 weeks gestation [including previous CS delivery]).

Another variable was the HDI, which, because of its simplicity, provides an overview of the region's development status, combining information on people's health, education, and income in a single number. This index has been used as a reliable comparative tool for public debate on the inequalities and priorities of the population.^[24]^

### 2.2 Statistical analysis

The data from each RG were statistically analyzed by considering the whole country and the states and cities with the highest and lowest HDI. These data were as follows: absolute total number of LBs, relative number of LBs by group (expressed as a percentage), absolute number of LBs by CS, CS rate by group, and relative contribution of each group to the CS rate (expressed as a percentage).

The *Z*-test for the comparison of proportions was used to evaluate the difference in the rate of LBs by CS among the state/city groups according to HDI. The *Z*-test statistic was used to determine if the difference between the sample and population means were statistically significant for large samples.

### 2.3 Ethical considerations

This study was approved by the research ethics committee through the unified national database of research records, *Plataforma Brasil* (number 3,492,807). The requirement for obtaining informed consent from the patients was waived because information collected was from secondary data sources.

## 3 Results

Between 2014 and 2017, a total of 11,774,665 LBs were reported in Brazil; of these, 6,580,432 (55.8%) were delivered through CS. During the 4-year study, RG5 (multiparous women with previous CS delivery) had the highest LB and CS absolute numbers and the highest contribution to global CS rate among all groups, increasing from 29.2% in 2014 to 33.3% in 2017 (Tables 1 and 2).

**Table 1:**
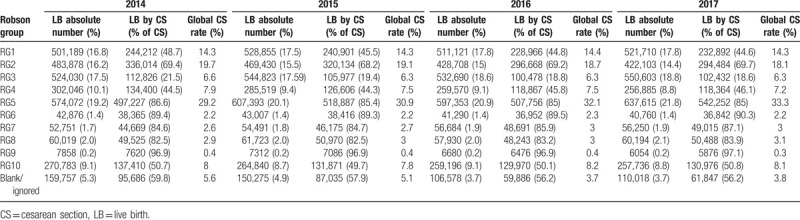
Robson classification of live births in Brazil, 2014–2017.

**Table 2:**
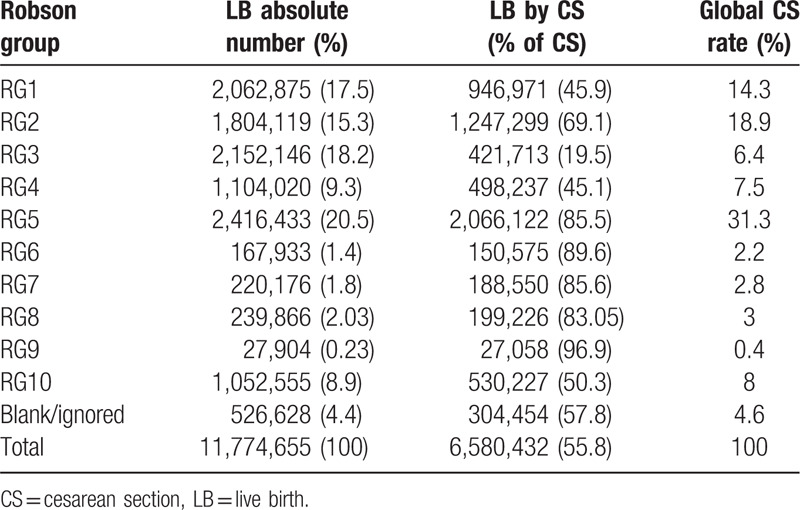
Robson classification of total live births in Brazil 2014–2017.

Brazil's HDI was 0.778 in 2017, which is within the range of high human development, but the HDI varied among Brazilian states. Alagoas, Maranhão, Pará, and Piauí had the lowest HDI in Brazil (0.600–0.699), whereas the Federal District, Santa Catarina, and São Paulo had the highest HDI (above 0.800).^[25]^ However, these differences increased in the inter-city comparison. Brazil has 44 cities with very high HDI values (range, 0.800–1.000) and 32 cities with very low HDI values (range, 0.000–0.499).^[26]^

An LB survey based on HDI conducted in 2014 to 2017 used RG classification to compare states and cities with the highest and lowest HDI values. The states with a high HDI had 3,034,782 LBs and a 59.3% CS rate, whereas the states with a low HDI had 1,416,357 LBs and a 48.7% CS rate. The cities with a high HDI had 1,757,434 LBs and 56.8% CS rate, and the cities with a very low HDI had 48,772 LBs and a 21.2% CS rate.

In Brazil, 60.2% of the population belong to RG 1 to 4 and represent 47.1% of all CS deliveries. RG5 represents 20.5% of LBs and 31.3% of all CS deliveries. RG 6 to 10 represents 14.4% of LBs and 16.4% of all CS deliveries.

RG5 (women with a previous full-term CS) contributed to the highest percentage of CS deliveries in Brazil (31.3%) and showed the greatest increase in the 4-year study compared to the other groups, in terms of the absolute CS number. The CS number of RG5 increased annually by 1% (mean value), from 29.2% in 2014 to 33.3% in 2017. The other groups had slightly decreased or maintained their absolute CS number over time. RG5 also had the highest CS global rate regardless of the region's HDI. States and cities with a high HDI had 33.7% and 32.7% of all CS deliveries in RG5, respectively, whereas states and cities with a low HDI had 28.03% and 24.4% of all CS deliveries in RG5, respectively.

RG10 (all women with a single cephalic pregnancy at <37 weeks’ gestation) represented 8.9% of LBs in Brazil, with a CS rate of 50.3%, contributing to 8% of the global CS rate. There were no significant changes in the group size or CS rates between 2014 and 2017. The size of the group presented no significant difference in HDI among regions, being slightly higher in states with a low HDI (9.8%) and cities with a very low HDI (8.8%).

Results published on the SINASC website also showed that women not assigned to any of the 10 RGs were classified as “blank and/or ignored.” In Brazil, 4.6% of pregnant women were not classified, and this accounts for 4.5% of the global CS rate. However, the states and cities with a high HDI presented lower percentages of “blank and/or ignored,” at 1.6% and 1.1%, respectively. These percentages were higher in the states and cities with a low HDI, at 7.9% and 14.3%, respectively.

In the general population, states and cities with a high HDI had a higher percentage of CS deliveries than those with a low HDI. The only exception to this trend occurred with those in RG9 (Tables 3 and 4).

**Table 3:**
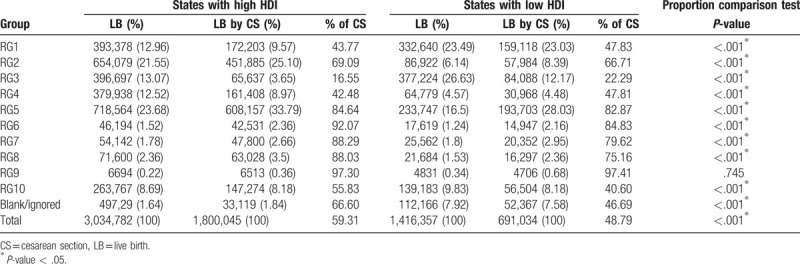
Robson classification of live births in states according to human development index.

**Table 4:**
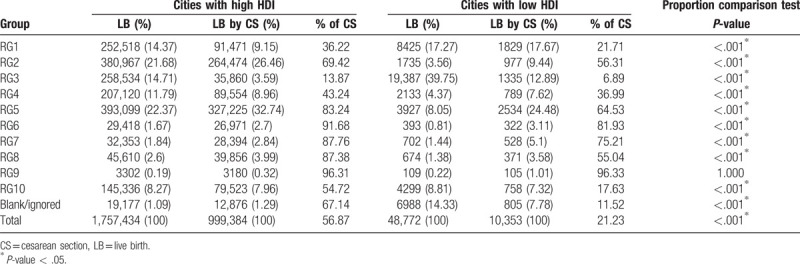
Robson classification of live births in cities according to human development index.

## 4 Discussion

This study reported and analyzed the CS rates in Brazil and compared the CS rates between regions with a high or low HDI using the Robson classification system.

CS rates in RG 1 to 4 are high in Brazil, although it should be low in these groups since they include nulliparous or multiparous women at term and without previous CS. At the other end of the scale, RG 6 to 10 was expected to have higher rates of CS because this group represents women with multiple noncephalic and premature pregnancies.^[27]^

States and cities with a high HDI present significantly higher CS rates than those with a low HDI. This study found a significant difference between RG1 (nulliparous women at term with spontaneous labor) and RG2 (nulliparous women at term with induced labor or delivery by CS) in terms of HDI, in the studied regions. The CS rate in RG1 was lower in regions with a high HDI than in regions with a low HDI. However, in RG2, an inverse association was observed, with the rate and total number of CS deliveries being higher in regions with a high HDI than in regions with a low HDI.

These data are consistent with those of another study conducted in Brazil by Nakamura-Pereira et al, who reported that RG2 is larger in private sector services and RG1 is larger in the public sector.^[28]^ This study also reported that 82.4% of women using the private health sector did not go into labor. This may explain the difference between regions in the present study, which demonstrated that RG2 is larger in regions with a high HDI, where there is better economic development and the population uses the private health sector (supplementary health) more than the public sector. In such areas, the CS rates are almost 70%. In Brazil, labor induction is not commonly used in the public or private sectors, and there is a preference for CS, without labor, in order to terminate pregnancy.^[29]^ Thus, it is assumed that most of CSs in RG2 were performed without labor.

RG1 and RG2 had the highest percentages of LBs in Brazil, except in cities with a very low HDI, where RG3 (multiparous women without previous CS and with spontaneous labor at >37 weeks’ gestation) had a larger percentage of LBs. These findings are consistent with those of a study conducted by the WHO, including 21 countries in which RG1 and RG2 were larger in areas with a high HDI, and RG3 was larger in areas with a very low HDI.^[10]^

Brazil has cesarean rates statistically higher than several countries, both when comparing the global rates, and in specific groups, such as groups 1 and 2 (Tables 5–7).^[30–32]^ When comparing the population formed by RG1 and RG2 between Brazil and other countries, they proved to be similar. ^[31,32]^ However, CS rates in groups 1 and 2 are statistically higher in Brazil than in the United States of America (USA) (Table 6).^[31]^ The CS rates in group 1 are statistically higher in Brazil than in Peru (Table 7).^[32]^ The difference is more evident when Brazil is compared to nations with low total CS rates (17%), such as Nordic countries.^[30]^ The CS rate is at least 3 times higher in Brazil than in these locations, which ranges from only 12.6% in Iceland to 16.8% in Finland.^[30]^ High CS rates in Brazil have no clinical bases^[18]^; rather, the labor and delivery care affects the delivery choices.^[33]^ For example, the high rate of CS in primiparous adolescents is not influenced by medical indication but by prenatal care and socioeconomic factors.^[34]^

**Table 5:**
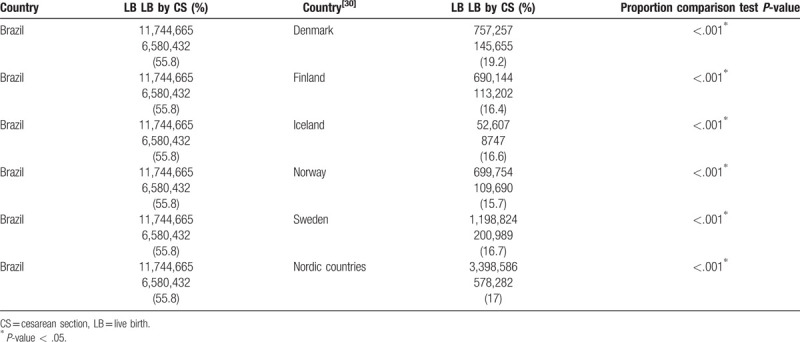
Comparison of cesarean rate between Brazil and Nordic countries.

**Table 6:**
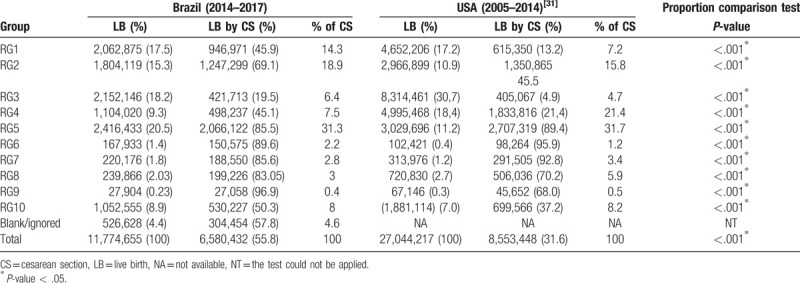
Comparison of cesarean rate between Brazil and USA.

**Table 7:**
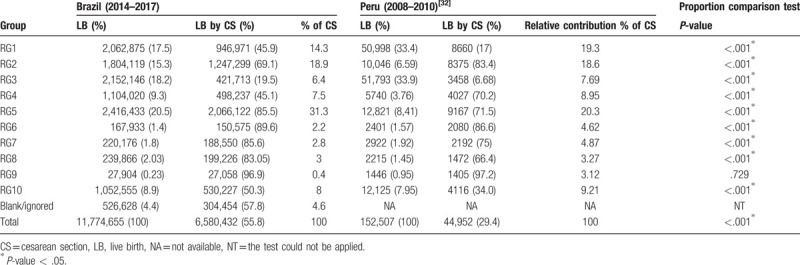
Comparison of cesarean rate between Brazil and Peru.

RG9 is the only group whose CS rate did not differ significantly between states and cities according to HDI. This result is expected because this group consists of women with different criteria to the Robson classification, defined by a transverse or oblique lie presentation rather than by mode of delivery.^[22]^ Research shows that the lower the percentage of CS delivery in GR9, the poorer the data collection quality.^[10]^ Likewise, the only RG in which there was no statistical difference in cesarean rates between Brazil and Peru was RG9, reaffirming the characteristics of this group (Table 7).^[32]^

Brazil has a higher RG10 birth percentage (all women with a single cephalic pregnancy at <37 weeks’ gestation, including previous CS) than the USA (7%),^[31]^ but it had a similar percentage to Peru (7.95%), another Latin American country.^[33]^ The CS rate in RG10 in Brazil is statistically higher than that in USA (37.2%) and Peru (34%) (Tables 6 and 7).^[31,32]^

In Brazil, 4.6% of pregnant women were not classified in any of the 10 RGs, reaching 14.3% in cities with a low HDI. These percentages are higher compared to those of countries with high HDI, such as the USA and Nordic countries,^[30,31]^ and to those reported in another study conducted in Brazil using prospective data.^[28]^ The WHO states that all women admitted for delivery in any facility can be classified into 1 of the 10 groups.^[35]^ Therefore, the proportion (14.3%) of nonclassified women in regions with a very low HDI may indicate a poor healthcare quality in these regions.

RG5 had the largest growth in the absolute number of CS over the 4-year study, with a mean increase of 1% per year. In another study conducted in Brazil between 2011 and 2012, RG5 presented the second highest CS rate.^[28]^ These data suggest that the number of women in RG5 has been increasing in Brazil since the beginning of the current decade. In the USA, RG5 presents the highest CS rate that, similarly to Brazil, has been increasing in recent years.^[31]^ Even in countries with lower CS rates, when RG5 increases, CS rates also increase.^[30]^ Furthermore, when the group size decreases, the total CS rate also decreases.^[30]^ The WHO reported that when RG5 exceeds 15%, the CS rate will usually be high.^[35]^

RG5 (women with previous CS at >37 weeks’ gestation) represents the largest group of LBs and has the highest contribution of global CS rate in the country. Both vaginal birth after CS (VBAC) and elective CS are known to be associated with risks and benefits; however, there are no large randomized studies that provide comparative data between both.^[36]^ Even though scientific evidence indicating that women who deliver through CS, with only an anterior transverse hysterectomy, have a lower risk of uterine rupture,^[37,38]^ reports of adverse results^[38,39]^ have decreased the tendency of doctors to encourage VBAC.^[40]^ The cause of the decline in the total rate of VBAC in recent decades^[40]^ is unclear, although the fear of legal medical disputes plays a role in this scenario.^[40–42]^

CS rates in RG5 are high in most countries, showing that VBAC is uncommon. The rate of VBAC significantly increased in the USA from 3% in 1981 to 31% in 1998.^[43]^ However, concerns about maternal and perinatal morbidity, risks of adverse perinatal outcome, women's misinformation, and increased medical litigations resulted in substantially decreased VBAC rates,^[29,44–46]^ which currently represent only 1.3% of all vaginal deliveries in the USA.^[31]^ Thus, these data indicate the difficulty in reducing CS rates, considering that women who have undergone a previous CS have a risk of adverse perinatal events. Although low, these factors may result in legal insecurity, eventually increasing the CS rates in this specific group of women.

### 4.1 Strengths and limitations

The strength of this study is that we analyzed the data of >11 million LBs in Brazil in 2014 to 2017. To the best of our knowledge, this is currently the largest epidemiological study to date in Brazil. Additionally, this study represents all Brazilian regions and uses the Robson classification system, a reliable and easy-to-use tool, to analyze and compare CS rates that can assist the creation of public policies to improve care during labor and delivery. Finally, the study provides a data platform to identify and analyze subgroups of individual patients, which may change practices and improve the global CS rates.

This study has a few limitations. First, we did not consider data from RG subdivisions, as SINASC does not provide this type of information. The WHO mentions some subdivisions in the Robson classification, for example, to separate induced delivery and CS delivery without labor, in RG2 and in patients with one or more previous CS deliveries in RG5. Second, we utilized a non-standardized collection of data on the Robson classification, which involved secondary data collected throughout the country by different professionals without a revision.

## 5 Conclusion

In Brazil, even pregnancies that present favorable conditions for vaginal delivery have high CS rates. In the general population, states and cities with a high HDI had a higher percentage of CS deliveries than those with a low HDI. The sizes of the nulliparous RG1 and RG2 were similar to those of countries with low CS rates. However, in Brazil, the CS rates in these 2 groups were higher, creating a cycle that increases RG5, consequently increasing the total CS rate. RG5 is the largest group that showed a constant and linear growth of 1% per year in its contribution to global CS rate. We suggest the implementation of healthcare policies to avoid CS as the first delivery method in nulliparous women, to address the high CS rates in Brazil.

## Acknowledgments

The authors would like to thank Editage (www.editage.com) for English language editing and H0consultoria (www.h0consultoria.com) for statistical services.

## Author Contributions

**Conception or design of the work**: Edson Luciano Rudey

**Data collection:** Edson Luciano Rudey

**Data analysis and interpretation:** Edson Luciano Rudey

**Drafting the article:** Edson Luciano Rudey

**Critical revision of the article:** Edson Luciano Rudey, Maria do Carmo Leal, Guilhermina Rego

**Final approval of the version to be published:** Edson Luciano Rudey, Maria do Carmo Leal, Guilhermina Rego

Edson Luciano Rudey orcid: 0000-0002-2834-2382.

Edson Luciano Rudey orcid: 0000-0002-2834-2382.
